# Prediction of BRCA gene mutation status in epithelial ovarian cancer by radiomics models based on 2D and 3D CT images

**DOI:** 10.1186/s12880-021-00711-3

**Published:** 2021-11-26

**Authors:** Liu Mingzhu, Ge Yaqiong, Li Mengru, Wei Wei

**Affiliations:** 1 Division of Life Sciences and Medicine, The First Affiliated Hospital of University of Science and Technology of China, University of Science and Technology of China, Hefei, 230031 Anhui China; 2 GE Healthcare China, Pudong New Area, No.1 Huatuo Road, Shanghai, 210000 China; 3 Department of Radiology, The First Affiliated Hospital of University of Science and Technology of China, Hefei, 230031 Anhui China; 4 Department of Radiology, Division of Life Sciences and Medicine, The First Affiliated Hospital of USTC, University of Science and Technology of China, Hefei, 230001 Anhui China

**Keywords:** Ovarian cancer, BRCA gene, Radiomics, Mutation

## Abstract

**Background:**

The objective of this study is to explore the value of two-dimensional (2D) and three-dimensional (3D) radiomics models based on enhanced computed tomography (CT) images in predicting BRCA gene mutations in patients with epithelial ovarian cancer.

**Methods:**

The clinical and imaging data of 106 patients with ovarian cancer confirmed by surgery and pathology were retrospectively analyzed and genetic testing was performed. Radiomics features extracted from the 2D and 3D regions of interest of the patients’ primary tumor lesions were selected in the training set using the maximum correlation and minimum redundancy method. Then, the best features were selected through Lasso tenfold cross-validation. Feature subsets were employed to establish a radiomics model. The model’s performance was evaluated via area under the receiver operating characteristic curve analysis and its clinical validity was assessed by using the model’s decision curve.

**Results:**

On the validation set, the area under the curve values of the 2D, 3D, and 2D + 3D combined models were 0.78 (0.61–0.96), 0.75 (0.55–0.92), and 0.82 (0.61–0.96), respectively. However, the DeLong test P values between the three pairs of models were all > 0.05. The decision curve analysis showed that the radiomics model had a high net benefit across all high-risk threshold probabilities.

**Conclusions:**

The three radiomics models can predict the BRCA gene mutation in ovarian cancer, and there were no statistically significant differences between the prediction performance of the three models.

## Introduction

According to data from the International Cancer Research Center, approximately 52,000 new cases of ovarian cancer are diagnosed in China each year, with a high tumor mortality rate [1, 2], and epithelial ovarian cancer accounts for 90% of all ovarian malignancies [3]. Approximately 50% of epithelial ovarian cancers exhibit DNA repair defects through homologous recombination, and BRCA1/2 mutations in germline and somatic cells are the most common mechanism underlying homologous recombination deficiency [4]. Patients with BRCA1/2 mutant ovarian cancer are sensitive to platinum drugs, have a higher progression-free survival period, and have better prognosis [5, 6]. The main method for clinical judgment of BRCA gene mutations is genetic testing at present, but it is costly and time-consuming, and unit sampling cannot cover the entire tumor [7]. Nougaret et al. [8] predicted BRCA gene status by observing CT features and found that some radiomics features are related to gene mutation status, but the judgment of these imaging features was dependent on the observer’s subjective experience.

Radiomics can objectively quantify the relationship between the pixels and the spatial distributions of medical images and fully explore the hidden information in the images that cannot be observed by the naked eyes. In previous studies, radiomics has been used to capture tumors’ inherent heterogeneity and correlate it with potential gene expression types [9]. Extracting radiomics features requires delineating the region of interest (ROI) of the lesion, which is currently done by two commonly used methods: two-dimensional (2D) and three-dimensional (3D) delineation. However, the advantages and disadvantages of these two delineation methods are still controversial [10, 11]. Therefore, in this study, we explored the predictive value of radiomics models based on different delineation methods for BRCA gene mutations in patients with epithelial ovarian cancer, and compared the prediction performance of each model, to improve the prediction of gene mutation status. In doing so, we strived to seek a model that could provide an ideal and convenient method to sketch the area of interest.

## Methods

### Research subjects

**Fig. 1:**
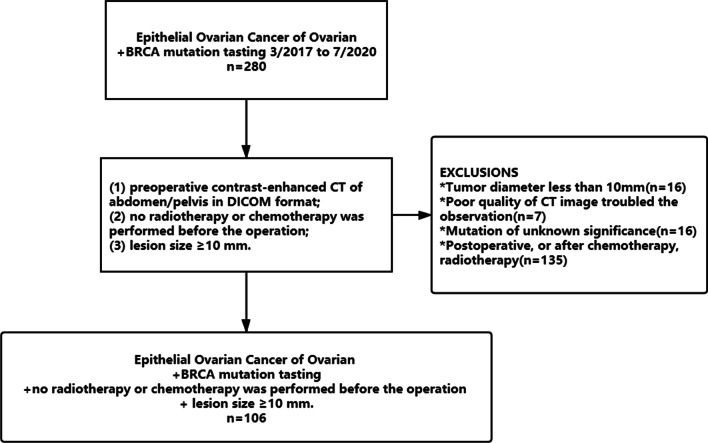
Flow chart of screening and grouping the enrolled cases

We performed a retrospective analysis of patients with epithelial ovarian cancer who received treatment at our institution from March 2017 to July 2020. We collected clinical pathological data, CT enhanced images before surgery, radiotherapy, and chemotherapy, and postoperative pathological diagnosis. The inclusion criteria were as follows: (1) epithelial ovarian cancer pathologically confirmed by biopsy; (2) abdominal CT scan performed before the operation, and the images including the arterial, venous, and delayed phases; (3) no radiotherapy or chemotherapy performed before the operation; (4) lesion size ≥ 10 mm. The exclusion criteria were as follows: (1) large image artifacts interfering with observation; (2) intolerance to enhanced CT examination; (3) preoperative radiotherapy and chemotherapy; (4) a history of other malignant tumors or pelvic metastases. In total, 106 patients were included in the study (Fig. 1). Among them, 63 cases, aged 35–75 years (average: 54.86 years), were in the BRCA gene non-mutation group; 43 cases aged 36–77 years (average: 53.93 years), were in the BRCA gene mutation group.

### Collection of general characteristics

The clinical data of 106 patients, including patient’s age, maximum tumor diameter and tumor markers, were collected and analyzed,.

### Genetic testing

NGS genetic testing technology was applied on all patients, who had at least one of the following indications: (1) family history of pathogenic BRCA gene mutations; (2) family history of breast cancer before the age of 45 years or triple-negative breast cancer before the age of 60 years; (3) affordability of genetic testing or the doctor’s requirement of genetic testing. To ensure the consistency of the test results, all subjects were tested by the same testing agency (Huada BGI).

### Image acquisition

The patients fasted for more than 8 h and drank approximately 500–1000 mL of clean water orally 15–30 min before the examination. After the bladder was filled, the abdomen and pelvis were scanned using a GE Discovery CT 750HD (HDCT, USA) scanner in supine position. The scan ranged from the top of the diaphragm or the level of the iliac spine to the symphysis pubis. The tube voltage was 120 kV, the tube current was 280–300 mA, the layer thickness was 5 mm, the reconstruction dimension was 1.25 mm, the layer spacing was 5 mm, and the pitch was 1.375:1. The contrast agent for enhanced scanning was 1.5 mL/kg of iohexol (300 mgI/mL), which was injected through the median cubital vein with a flow rate of 2.5–3.0 mL/s. The contrast agent was injected twice: at 25–30 s and 60–70 s for arterial and venous scanning, respectively.

### Image segmentation and feature extraction

**Fig. 2:**
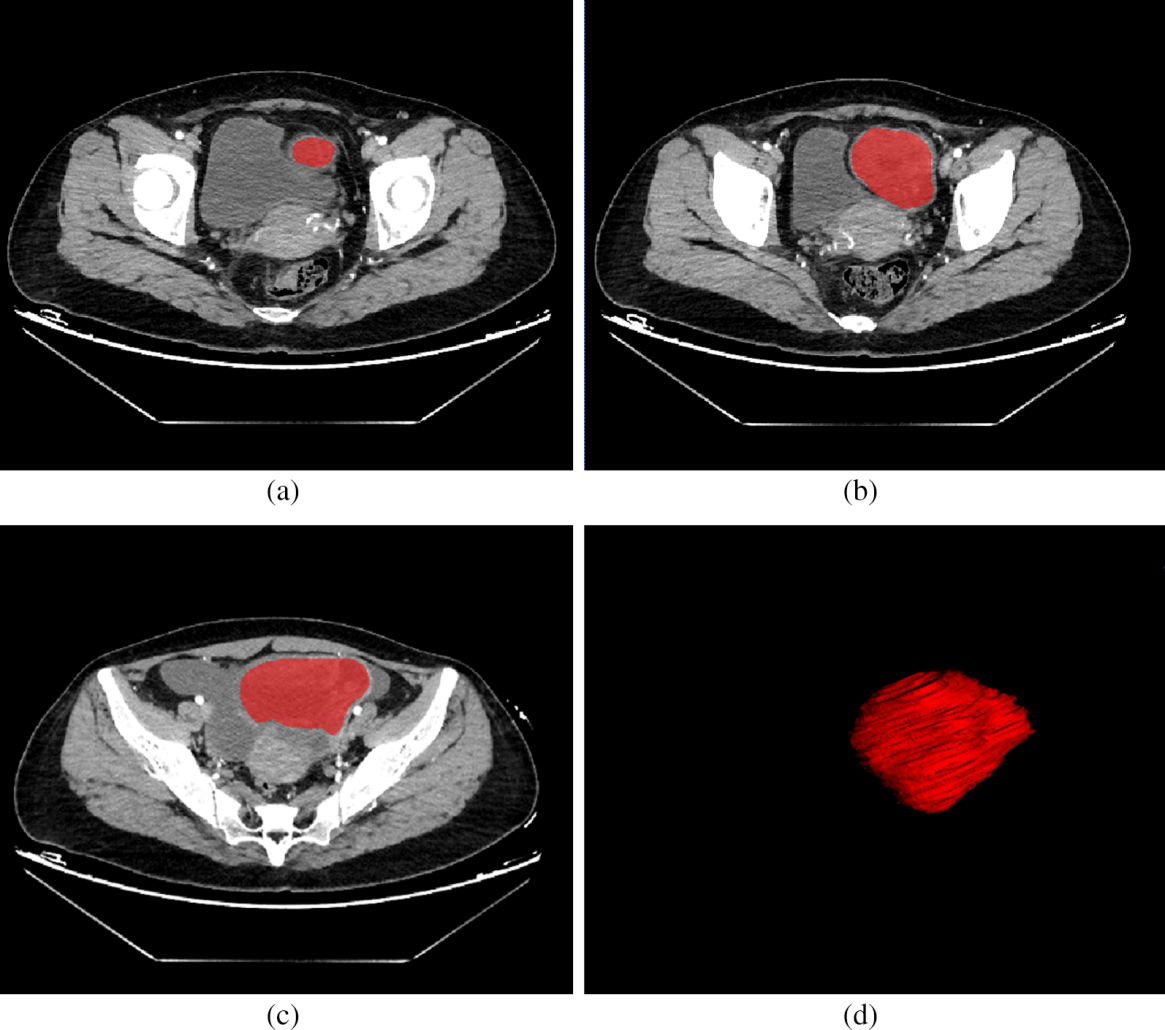
**a–**-**d** Images of a 35-year-old patient with high-grade (stage IIIC) serous carcinoma of the ovary, which was genetically detected with a non-BRCA gene mutation. ITK-SNAP software was used to segment and label the lesion layer-by-layer to generate a 3D image of the lesion

The preoperative abdominal enhanced CT image data of all patients were collected from the ICPACS workstation of the CT room at the Imaging Department of our institution and exported in.DICOM format. For all the image modality, the slices were resampled to 1*1*1cm^3^, and the intensity range were normalized to a mean value of 0 and standard deviation of 1 (z score standardization). Doctor A, a senior diagnostic imaging doctor, used ITK-Snap software (https://www.itksnap.org) to delineate the (ROI) of the lesion in the third phase of enhanced CT. For 2D delineation, the scope included the layer with the largest lesion surface area, and for 3D delineation, the scope covered all possible areas of the target lesion. During the delineation, necrosis, blood vessels, and other structures were eliminated as completely as possible (Fig. 2). Two weeks later, doctor A randomly selected data of 30 patients to perform ROI delineation again to evaluate the intra-group consistency of ROI delineation (intra-ICC). To evaluate the between-group consistency of ROI delineation (inter-ICC), another radiologist, B, also drew the ROIs on the 30 patients’ images independently.

A.K. software (GE Healthcare, AnalysisKit; Version: 3.2.0.R) conforming to the IBSI standard was used to extract features from the ROIs outlined in each image. A total of 681 image biomarkers were obtained in each phase (non-mutation group labeled PA1-63, marked as 0; mutation group labeled PA64-106, marked as 1), including histogram, grayscale interconnected area matrix, grayscale co-occurrence matrix, morphology, run-length matrix, and wavelet change (wavelet) and Laplacian change (ln) characteristics.

### Feature screening and model establishment

The features of the arterial phase, venous phase, and delay phase were integrated into 2043 features. The data were randomly divided into the training and test sets in a 7:3 ratio. In the training set, maximum correlation minimum redundancy (mRMR) and LASSO (least absolute shrinkage and selection operator) tenfold cross-validation, were used to select features. First, mRMR was performed to remove redundant and irrelevant features, incorporate the retained features into the LASSO regression, find the value of hyperparameter λ that minimized the binomial deviation, and then retain the subset of features whose coefficients were not 0 to construct the final model.

### Statistical analysis and processing

The chi-square test or Mann–Whitney U test was used to check for significant differences in general features and clinical characteristics between the two groups. The area under curve (AUC), model accuracy, precision, sensitivity, specificity, negative predictive value, and positive predictive value were analyzed to evaluate the radiomics models’ predictive ability. Finally, decision curve analysis was used to evaluate the models’ clinical applicability. All statistical analyses were performed using R software (version 3.6.1, https://www.r-project.org).

For general clinical characteristics, we used the Chi-square test and the Kruskal–Wallis H test to analyze abnormally distributed continuous variables. P values < 0.05 were considered statistically significant. All statistical analyses were performed using R software (version 3.6.1) and the Python programming language (version 3.5.6).

The intraclass correlation coefficients (ICCs) of each texture feature were calculated to evaluate the within- and between-observer changes in the texture features extracted by the ROI segmentation and to explain each feature’s reproducibility, according to the following scale: ICC < 0.4, poor; 0.59 > ICC ≥ 0.4, fair; 0.75 > ICC ≥ 0.6, good; and ICC ≥ 0.75, excellent. Features with ICC ≥ 0.8 were considered stable and included in further analyses.

## Results

### General characteristics

No statistically significant differences in age composition, maximum tumor diameter, or tumor markers were found among the three groups (*P* > 0.05; Table 1).

**Table 1:** General characteristics of the three groups

Variable	Sample	Class meaning unknown	Class mutation	Class wild	Statistics	*P *value
Age	106	55.00 (49.00, 58.05)	52.00 (47.70, 57.20)	54.00 (48.00, 59.30)	0.781	0.677
Maximum_Diameter	106	8.75 (5.95, 11.00)	7.00 (4.57, 9.24)	7.00 (4.49, 10.00)	2.929	0.231
Tumor marker CA125	86	15 (83.33%)	42 (93.33%)	63 (81.82%)	3.601	0.463
Tumor marker CA125、CA199	19	3 (16.67%)	3 (6.67%)	13 (16.88%)		
Tumor marker CA199	1	0 (0.00%)	0 (0.00%)	1 (1.30%)		

### Construction and verification of prediction models

#### Feature screening

The ICC ranges within and between the 2D-outlined feature data groups were (− 0.18–1.00) and (− 0.08–1.00), respectively. In total, 531 features had ICC values of > 0.75 across the two groups. In the 3D-outlined feature data group, the ICC ranges within and between groups were (− 0.06–1.00) and (− 0.40–1.00), respectively. In total, 548 features had ICC values of > 0.75 across the two groups.

**Fig. 3:**
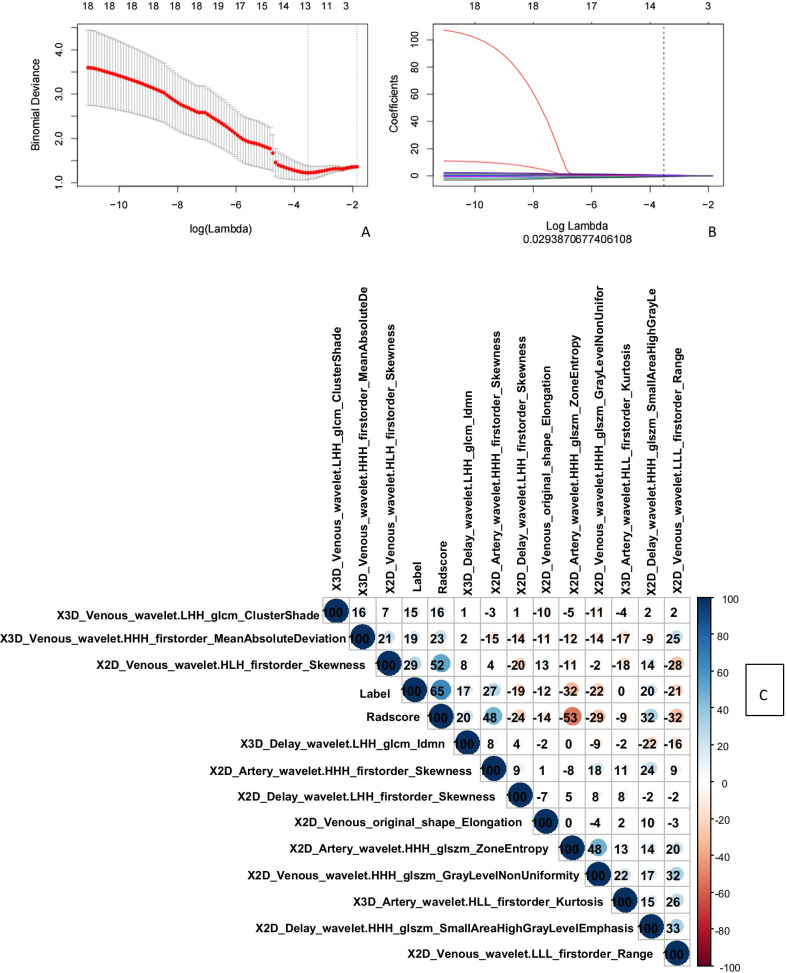
Application of LASSO (Least absolute shrinkage and selection operator)-logistic regression to imaging feature screening in the 2D + 3D model shows that the LASSO-logistic regression model selects tuning parameters (λ) through tenfold cross-validation and obtains the relationship between binomial variance and logarithm (λ) (**a**). The relationship is retained with the parameters that yield the smallest binomial deviation, and the 12 best features with non-zero coefficients (**b**) are retained in the final model. The relationships between the features and gene mutation status (correlation coefficient × 100) are shown in the heat map (**c**)

We then used the mRMR and LASSO regression models to perform feature screening on features with ICC values of > 0.75 in the 2D, 3D, and 2D + 3D models. For the 2D images, the binomial deviation was smallest at the optimal tuning parameter value of ln λ = 0.0118, and the four features whose coefficients were not 0 at that value were retained. For the 3D images, the binomial deviation was smallest at the optimal tuning parameter value of ln λ = 0.0279, and the 13 features whose coefficients were not 0 at that value were retained. For the 2D + 3D images, the binomial deviation was the smallest at the optimal tuning parameter value of ln λ = 0.0294 (Fig. 3a), and the 12 features whose coefficients were not 0 at that value (Fig. 3b) were retained; eight and four were retained from 2 and 3D images, respectively. The corresponding coefficients of the features are shown in Fig. 3c.

#### Diagnostic efficiency of the devised models

Logistic regression was used to establish radiomics models using the selected features, and the radscore of each case was calculated. Table 2 shows the radscore distribution in the gene mutation and wild-type groups generated by the 2D, 3D, and 2D + 3D models. We used the AUC of the receiver operating characteristic curve and the models’ accuracy, sensitivity, specificity, negative predictive value, and positive predictive value to evaluate their predictive ability.

**Table 2:** Radscores of each patient in the training and validation sets calculated by different models, and their distribution and difference statistics in patients with wild-type and mutant type BRCA genes

	Variable	Sample	Wild	Mutation	Statistics	*P* value
Train	Radscore _2D3D	76	− 1.22 ± 1.06	0.52 ± 0.94	− 7.329	< 0.001
	Radscore _3D	76	− 0.71 (− 1.06, − 0.28)	0.00 (− 0.36, 0.28)	− 4.413	< 0.001
	Radscore _2D	76	− 0.51 ± 0.23	− 0.19 ± 0.30	− 5.303	< 0.001
Test	Radscore_2D3D	30	− 1.03 (− 2.32, − 0.01)	0.66 (− 0.23, 4.92)	− 2.963	0.003
	Radscore_3D	30	− 0.61 (− 0.95, − 0.25)	− 0.04 (− 0.48, 0.49)	− 2.159	0.031
	Radscore_2D	30	− 0.41 ± 0.22	− 0.17 ± 0.23	− 2.9	0.007

**Fig. 4:**
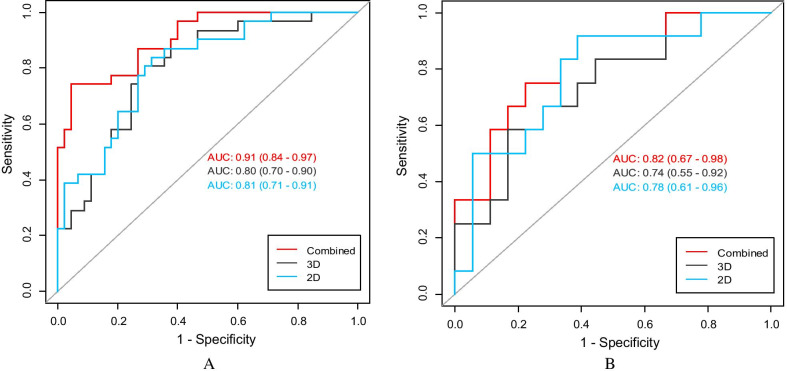
ROC curves of 2D model, 3D model, and 2D + 3D model in the training group (**a**) and validation group (**b**)

In the training set, the 2D model’s image AUC was 0.81 (0.71–0.91), the 3D model’s image AUC was 0.80 (0.70–0.90), and the 2D + 3D model’s image AUC was 0.91 (0.84–0.97). In the test set, the 2D model’s image AUC was 0.78 (0.61–0.96), the 3D model’s image AUC was 0.75 (0.55–0.92), and the 2D + 3D model’s image AUC was 0.82 (0.67–0.98) (Table 3). The highest AUC value of the 2D + 3D model indicated its highest diagnostic efficacy (Fig. 4). However, the DeLong test results showed that the P values among the three pairs of models were all > 0.05, suggesting that there were no statistically significant differences in prediction efficiency among the three pairs of models.

**Table 3:** Cutoff value prediction ability of the 2D, 3D, and 2D + 3D joint radiomics models

	AUC	Accuracy	Sensitivity	Specificity	Positive value	Negative value	Cut-off
2D Training set	0.81 (0.71–0.91)	0.75 (0.63–0.84)	0.68	0.83	0.86	0.65	− 0.43
2D validation set	0.78 (0.61–0.96)	0.73 (0.54–0.87)	0.61	0.91	0.91	0.61	
3D training set	0.80 (0.70–0.90)	0.75 (0.63–0.84)	0.71	0.81	0.84	0.65	− 0.61
3D validation set	0.75 (0.55–0.92)	0.74 (0.55–0.88)	0.84	0.58	0.76	0.70	
2D + 3D Training set	0.91 (0.84–0.97)	0.86 (0.77–0.93)	0.95	0.74	0.84	0.92	0.14
2D + 3D validation set	0.82 (0.67–0.98)	0.73 (0.54–0.87)	0.611	0.91	0.91	0.61

**Fig. 5:**
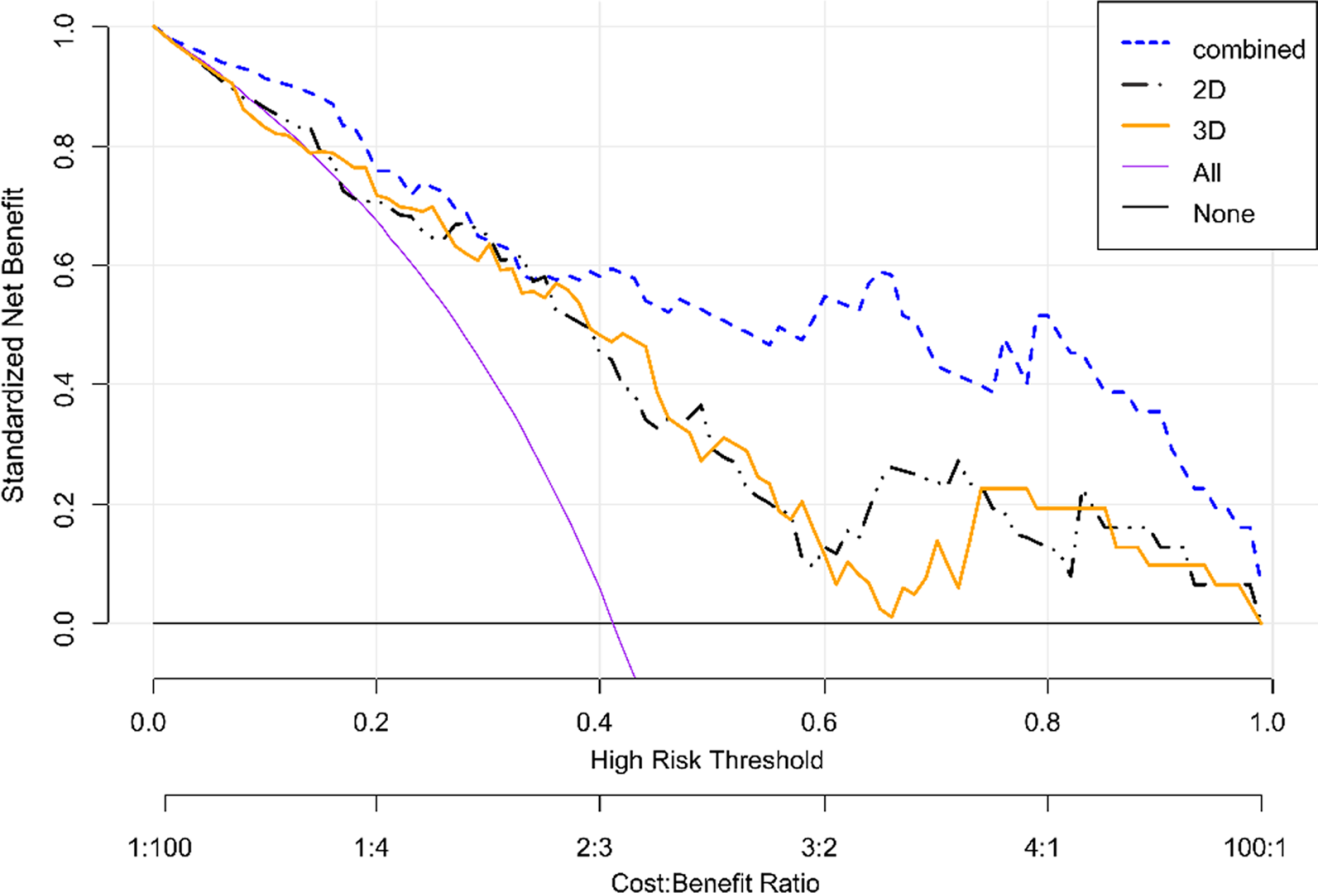
The yellow line, black dotted line, and blue dotted line represent the data obtained from the 2D, 3D, and 2D + 3D images, respectively. The x-axis represents the patient’s personal threshold probability (e.g., x = 0.6 means that the high-risk threshold of ovarian cancer and BRCA gene mutation is 60%). The y-axis represents net income. The line labeled “All” represents the hypothesis that all ovarian cancer cases are caused by BRCA gene mutations. The thin line labeled “None” represents the assumption that there are no BRCA gene mutations in patients with ovarian cancer

Finally, a decision curve was used to evaluate the three models’ clinical effectiveness (Fig. 5). As the radiomics model’s standard net benefit within a range of the high-risk threshold increased, its clinical effectiveness increased. The decision curves show that the clinically effective performance of the 2D + 3D model was much higher than those of the other two models.

The above results indicate that the radiomics model based on 2D and 3D images can effectively predict BRCA gene mutations in patients with epithelial ovarian cancer, although the 2D + 3D model showed no significantly different predictive performance from that of the radiomic models based on 2D or 3D images alone.

## Conclusions

Ovarian cancer has the highest mortality rate among gynecological malignancies. More than 70% of patients with ovarian cancer are already in the advanced stage when detected [12], and there is still a lack of accurate early diagnosis and prevention methods [13]. The use of radiomics can avoid the disadvantages of conventional imaging examinations such as subjectivity, histopathology, and local sampling.

Guo Jianlin et al. [14] found that the combined radiomics model of arterial phase and venous phase had higher predictive performance than the separate model of each phase. Using the CT features of 539 cases of lung adenocarcinoma, Yooh et al. [15] analyzed the tumor size, location, volume, density, CT value, and the relationship between pixel-based texture features and gene expression patterns and found that these image features showed good discrimination of fusion-positive from fusion-negative lung adenocarcinomas. In this study, the quantitative features were selected and the prediction model was established using radiomics features extracted from the arterial phase, venous phase, and delay phase of enhanced CT images, and the resulting radiomics scores were strongly correlated with the BRCA gene mutation (r = 0.65, P < 0.001; Fig. 3c). Totally, 2,043 radiomics features were extracted from the CT images of 106 patients with epithelial ovarian cancer, Twelve characteristics were finally selected and the model was constructed via multivariate logistic regression. Among them, the P values of the 3D_Artery_wavelet.HLL_firstorder_Kurtosis, 3D-Delay-wavelet.LHH_IDMN, Venous_wavelet.LLL_firstorder_Range, Delay_wavelet.HHH_glszm_SmallAreaHighGrayLevelEmphasis, Venous_wavelet.HHH_firstorder_MeanAbsoluteDeviation, and 2D_Artery_wavelet.HHH_glszm_ZoneEntropy were less than 0.05, and can be used as independent risk predictors. The values of these characteristics in the mutation group were higher than those in the non-mutation group. For example, Artery_wavelet.HLL_firstorder_Kurtosis is a measure of the “peak” of the value distribution in the image ROI transferred by wavelet from the artery phase. Higher kurtosis means that the quality of the distribution is concentrated in the tail instead of the average, which can be used as a label to predict the mutation status of the BRCA gene. Other detailed explanation of the features are in the appendix (Table 4). The radscore obtained from the mutlvirate logistic regression model was also higher in the mutation group than the wild group in both the 2D and 3D and 2D + 3D model(p < 0.05). Therefore, the radiomics signature established by the selected radiomics feature radscore can be used as a biomarker for BRCA gene mutation prediction.

**Table 4:** The detailed information of the features

Image type	Features	Features explanation
X3D-venous-wavelet.LHH	Glcm–cluster shade $\mathop \sum \limits_{i = 1}^{{N_{g} }} \mathop \sum \limits_{j = 1}^{{N_{g} }} \left( {i + j - \mu_{x} - \mu_{y} } \right)^{3} p\left( {i,j} \right)$	Cluster Shade is a measure of the skewness and uniformity of the GLCM. A higher cluster shade implies greater asymmetry about the mean
X3D-venous-wavelet.HHH	Firstorder –mean absolute deviation (MAD)$\frac{1}{{N_{p} }}\mathop \sum \limits_{i = 1}^{{N_{p} }} \left| {X\left( i \right) - X } \right|$	Mean Absolute Deviation is the mean distance of all intensity values from the Mean Value of the image array
X2D-venous-wavelet.HLH X2D-delay-wavelet.LHHX2D-Artery-wavelet.HHH	Firstorder—skewness $\frac{{\mu_{3} }}{{\sigma^{3} }} = \frac{{\frac{1}{{N_{p} }}\mathop \sum \nolimits_{i = 1}^{{N_{p} }} \left( {X\left( i \right) - X} \right)^{3} }}{{\left( {\sqrt {\frac{1}{{N_{p} }}} \sqrt {\mathop \sum \nolimits_{i = 1}^{{N_{p} }} \left( {X\left( i \right) - X} \right)^{2} } } \right)^{3} }}$	Skewness measures the asymmetry of the distribution of values about the Mean value. Depending on where the tail is elongated and the mass of the distribution is concentrated, this value can be positive or negative
X3D-delay-wavelet.LHH	Glcm—IDMN $\mathop \sum \limits_{k = 0}^{{N_{g} - 1}} \frac{{P_{x - y} \left( k \right)}}{{1 + \left( {\frac{{k^{2} }}{{N_{g}^{2} }}} \right)}}$	IDMN (inverse difference moment normalized) is a measure of the local homogeneity of an image. IDMN weights are the inverse of the Contrast weights (decreasing exponentially from the diagonal i = ji = j in the GLCM). Unlike Homogeneity2, IDMN normalizes the square of the difference between neighboring intensity values by dividing over the square of the total number of discrete intensity values
X2D-venous	Original shape elongnation $\sqrt {\frac{{\lambda_{minor} }}{{\lambda_{major} }}}$	Elongation shows the relationship between the two largest principal components in the ROI shape. For computational reasons, this feature is defined as the inverse of true elongation
X2D-venous-wavelet.HHH	Glszm–Zone entropy (ZE)$- \mathop \sum \limits_{i = 1}^{{N_{g} }} \mathop \sum \limits_{j = 1}^{{N_{s} }} p\left( {i,j} \right)\log_{2} \left( {p\left( {i,j} \right) + \in } \right)$	ZE measures the uncertainty/randomness in the distribution of zone sizes and gray levels. A higher value indicates more heterogeneneity in the texture patterns
X2D-venous-wavelet.HHH	Glszm–gray level non-uniformity (GLN)$\frac{{\mathop \sum \nolimits_{i = 1}^{{N_{g} }} \left( {\mathop \sum \nolimits_{j = 1}^{{N_{s} }} P\left( {i,j} \right)} \right)^{2} }}{{N_{z} }}$	GLN measures the variability of gray-level intensity values in the image, with a lower value indicating more homogeneity in intensity values
X3D-artery-wavelet.HLL	Firstorder—Kurtosis $\frac{{\mu _{4} }}{{\sigma ^{4} }} = \frac{{\frac{1}{{{\text{N}}_{{\text{p}}} }}\sum\nolimits_{{{\text{i}} = 1}}^{{{\text{N}}_{{\text{p}}} }} {\left( {{\text{X}}\left( {\text{i}} \right) - {\text{X}}} \right)^{4} } }}{{\left( {\frac{1}{{{\text{N}}_{{\text{p}}} }}\sum\nolimits_{{{\text{i}} = 1}}^{{{\text{N}}_{{\text{p}}} }} {\left( {{\text{X}}\left( {\text{i}} \right) - {\text{X}}} \right)^{2} } } \right)^{2} }}$	Kurtosis is a measure of the ‘peakedness’ of the distribution of values in the image ROI. A higher kurtosis implies that the mass of the distribution is concentrated towards the tail(s) rather than towards the mean. A lower kurtosis implies the reverse: that the mass of the distribution is concentrated towards a spike near the Mean value
X2D-delay-wavelet.HHH	Glszm–small area high gray level emphasis (SAHGLE)$\frac{{\mathop \sum \nolimits_{i = 1}^{{N_{g} }} \mathop \sum \nolimits_{j = 1}^{{N_{s} }} \frac{{P\left( {i,j} \right)i^{2} }}{{j^{2} }}}}{{N_{z} }}$	SAHGLE measures the proportion in the image of the joint distribution of smaller size zones with higher gray-level values
X2D-venous-wavelet.LLL	Firstorder–range max(X)-min(X)	The range of gray values in the ROI

The study by Lei Xu et al. found that in both univariate and multivariate analyses, 3D image features showed better prediction performance than 2D image features [16]. However, according to Shen et al. [17], 2D features had better performance. Yet, Lifeng Yang et al. [18] found that the features derived from a 2D + 3D model showed better prognostic performance than 2D or 3D features alone in predicting overall survival of non-small cell lung cancer. Therefore, we established prediction models using 2D, 3D, and 2D + 3D image features, and found that the AUC values of all three models were over 0.75, and all three models’ exhibited good prediction performance, indicating that the radiomics models can effectively predict BRCA gene mutation status in patients with epithelial ovarian cancer. The 2D + 3D model had the highest AUC value, but the DeLong test results showed no statistical differences in predictive performance between the three pairs of models. The results showed that 2D outlining of the layer with the largest lesion diameter could cover the central region of the tumor and achieve high predictive performance in predicting BRCA gene mutations in ovarian cancer. The 3D outlining images could provide information about tumors’ heterogeneity outside the central area, but this information could not contribute to the prediction of BRCA gene mutations in the present study. Therefore, we believe that when the image is delineated in 2D, with appropriate selection of lesion delineation level, the predictive performance the radiomics model is close to that of the 2D + 3D model. This insight may improve the practical efficiency of establishing radiomics model in clinical practice.

This study had some limitations. First, this study was a single-center retrospective study. The included cases were all from our hospital and therefore lacked external verification. Second, the sample size was small, which may lead to bias in the data. Third, all ROIs in this study were drawn manually, making the results easily affected by subjective factors. Lastly, in this study, there were 63 and 43 patients in the wild-type and mutant groups, respectively. The data of the two groups were unbalanced, and the model may therefore be biased.

In summary, the 2D, 3D, and 2D + 3D radiomics models based on enhanced CT images can effectively predict BRCA gene mutation status in patients with epithelial ovarian cancer and provide a new type of method for clinical evaluation of patients’ genetic mutation status.

## Data Availability

The datasets used and/or analyzed in the current study are available from the corresponding author on reasonable request.
